# Roles of Myokines and Muscle-Derived Extracellular Vesicles in Musculoskeletal Deterioration under Disuse Conditions

**DOI:** 10.3390/metabo14020088

**Published:** 2024-01-26

**Authors:** Jie Zhang, Yunfang Gao, Jiangwei Yan

**Affiliations:** 1 Institute of Special Medicine, Shanxi Medical University, Jinzhong 030619, China; zhangjie96@sxmu.edu.cn; 2 Shaanxi Key Laboratory for Animal Conservation, College of Life Sciences, Northwest University, Xi’an 710069, China

**Keywords:** disuse, extracellular vesicles, muscle–bone communication, musculoskeletal deterioration, myokines

## Abstract

Prolonged inactivity and disuse conditions, such as those experienced during spaceflight and prolonged bedrest, are frequently accompanied by detrimental effects on the motor system, including skeletal muscle atrophy and bone loss, which greatly increase the risk of osteoporosis and fractures. Moreover, the decrease in glucose and lipid utilization in skeletal muscles, a consequence of muscle atrophy, also contributes to the development of metabolic syndrome. Clarifying the mechanisms involved in disuse-induced musculoskeletal deterioration is important, providing therapeutic targets and a scientific foundation for the treatment of musculoskeletal disorders under disuse conditions. Skeletal muscle, as a powerful endocrine organ, participates in the regulation of physiological and biochemical functions of local or distal tissues and organs, including itself, in endocrine, autocrine, or paracrine manners. As a motor organ adjacent to muscle, bone tissue exhibits a relative lag in degenerative changes compared to skeletal muscle under disuse conditions. Based on this phenomenon, roles and mechanisms involved in the communication between skeletal muscle and bone, especially from muscle to bone, under disuse conditions have attracted widespread attention. In this review, we summarize the roles and regulatory mechanisms of muscle-derived myokines and extracellular vesicles (EVs) in the occurrence of muscle atrophy and bone loss under disuse conditions, as well as discuss future perspectives based on existing research.

## 1. Introduction

Space flight, prolonged bed rest, and a sedentary lifestyle typically lead to reductions in muscle mass, fiber cross-sectional area, and contractile force. Moreover, decreased glycolipid metabolism in skeletal muscle under disuse conditions can result in a series of metabolic syndromes, including obesity. Existing muscle atrophy can also significantly exacerbate bone loss, thereby increasing the risk of fracture, disability, and death. Therefore, understanding the mechanisms involved in disuse-induced musculoskeletal dysfunction can not only provide a scientific basis for effectively preventing muscle atrophy and bone loss, but can also prevent the development of metabolic syndromes.

Given the common origin of skeletal muscle and bone, as well as their structurally adjacent and functionally dependent characteristics, emerging studies have focused on concurrent muscle–bone research based on individual investigations of muscle and bone. Notably, previous studies have reported that skeletal muscle atrophy in mice can occur after one week of hindlimb unloading, while bone microstructure deterioration and bone mechanical weakening can occur after two weeks of hindlimb unloading [1]. Research on rats has shown a decrease in muscle mass after two weeks of hindlimb unloading, with a decline in bone mineral density and mechanical properties after four weeks of hindlimb unloading [2]. This evidence suggests that skeletal muscle changes occur earlier than bone alterations under disuse conditions [3], implying that skeletal muscle atrophy may be involved in mediating the occurrence of bone loss under disuse conditions.

Myokines represent a group of soluble molecules secreted by skeletal muscles, including irisin, myostatin, and others. Corresponding changes in myokines, especially their circulating and muscle concentrations, under mechanical unloading are closely related to disuse-induced skeletal muscle atrophy and bone deterioration [4,5]. In addition to myokines, other molecules, especially microRNAs (miRNAs), are encapsulated in lipid bilayers and secreted into the extracellular space, which are then recognized by recipient cells and participate in the regulation of biological functions [6]. Considering the protective effect of the lipid bilayer on its contents, extracellular vesicles (EVs) are also recognized as another highly regarded signal carrier [7]. Emerging evidence has reported corresponding changes in serum and muscle concentrations of muscle-derived EVs under disuse conditions [8]. Moreover, muscle-derived EVs are also involved in the disturbance of bone metabolism under disuse conditions [9]. In the present review, we update and summarize the regulatory roles and mechanisms of myokines and muscle-derived EVs in the development of musculoskeletal deterioration under disuse conditions. Our aim is to provide a comprehensive reference for the prevention of muscle atrophy and bone loss from the perspective of skeletal muscle-derived biochemical signals.

## 2. Roles of Myokines on Musculoskeletal Metabolism and Homeostasis under Normal and Disuse Conditions

Myokines are soluble molecules expressed and released by muscle fibers to regulate biological and pathological activities in local and distant organs, such as the skeletal muscle, bone, fat, and heart [10]. Approximately 672 myokines have been identified to date [11]. In this section, we focus on myokines essential for the maintenance of musculoskeletal health, such as irisin, myostatin, β-aminoisobutyric acid (BAIBA), lumican, and interleukin 6 (IL-6), detailing their changes and roles in the context of musculoskeletal deterioration under disuse conditions (Table 1).

### 2.1. Irisin

Irisin, derived from the proteolytic processing of fibronectin type III domain-containing 5 (FNDC-5), acts as an exercise-induced myokine to promote thermogenesis by browning white fat [48]. In addition to its indispensable roles in fat, emerging evidence has suggested that irisin plays a pleiotropic positive role in muscles throughout most developmental phases. In detail, irisin promotes satellite cell activation [12], enhances myoblast proliferation and fusion [13], maintains muscle protein balance by down-regulating protein degradation and up-regulating protein synthesis [12], stimulates muscle growth by up-regulating the mRNA expression of myokines that play positive roles in skeletal muscles, such as insulin-like growth factor 1 (IGF-1), while down-regulating the mRNA expression of myokines that play negative roles in skeletal muscles like myostatin [14], preserves muscle cells from senescence [15], promotes mitochondrial fusion, and increases mitochondrial content in skeletal muscles [16,17]. The positive regulatory effects of irisin on skeletal muscle are consistent with the occurrence of significantly enhanced grip strength following irisin administration [49].

Growing evidence has also demonstrated varying positive roles of irisin on bone health. Research has shown that irisin administration can enhance osteoblast proliferation and differentiation by promoting the mRNA expression of osteogenic markers, including alkaline phosphatase protein (ALP), collagen I (COL-1), runt-related transcription factor 2 (RUNX-2), osterix, osteopontin (OPN), osteocalcin (OCN), osteoprotegerin (OPG), and estrogen receptor alpha (ERα), which are likely induced by activating the P38/extracellular signal-regulated kinase (ERK)/mitogen-activated protein kinase (MAPK) signaling pathway [18,19,20,21,22,23]. Irisin also promotes osteogenesis by promoting macrophage M2 polarization and mediating anti-inflammatory effects by activating the AMP-activated protein kinase (AMPK) signaling pathway [24], and it is reported to enhance the mineralization of osteoblasts [19,22]. Irisin also regulates bone resorption and promotes osteoclast precursor cell proliferation via the P38 and c-Jun N-terminal kinase (JNK) signaling pathway but inhibits the differentiation of osteoclast cells by down-regulating the receptor activator of nuclear factor-κ-gene binding (RANK) and inhibiting the nuclear factor kappa-B (NF-κB) pathway [19,22,23,25]. Therefore, maintaining bone remodeling homeostasis may be a crucial mechanism by which irisin acts on bone health. Irisin is also involved in preventing osteocyte-like cell (MLO-Y4) apoptosis [26], underscoring its essential regulatory roles in bone maintenance. Overall, the above evidence highlights the global regulatory effects of irisin on bone homeostasis, consistent with the increases in cortical bone mass and strength after irisin administration [23]. Furthermore, the observed positive correlation between irisin concentration and bone mineral density in humans further emphasizes its beneficial role in bone health [50,51,52].

Given the positive impact of irisin on the musculoskeletal system, its changes and influences under mechanical unloading conditions have attracted considerable attention. Recent research revealed a reduction in serum levels of irisin in mice following four weeks of hindlimb unloading [15], accompanied by a decline in FNDC5 mRNA expression in hindlimb skeletal muscles after 3–4 weeks of hindlimb unloading [15,53], potentially linked to the down-regulation of the bone morphogenetic protein (BMP) and phosphatidylinositol 3-kinase (PI3K) signaling pathways [53]. In addition, a similar decline in irisin serum level has also been observed in young human males on the second day of recovery after 10 days of bed rest [54]. These findings suggest that irisin level could serve as a potential prognostic marker for disuse-induced musculoskeletal deterioration. Subsequent investigations have shown that irisin administration can counteract impairments in the musculoskeletal system induced by disuse. For instance, Colaianni et al. demonstrated that irisin mitigates muscle atrophy induced by hindlimb unloading and effectively prevents the reduction in myosin heavy chain (MHC) isoforms [15,55]. In addition, irisin treatment has been shown to alleviate bone loss induced by disuse by up-regulating the mRNA expression of osteogenic markers such as Alp, Col-1, and Opg, increasing calcium deposition to prevent primary osteoblast deterioration [55,56,57], and increasing osteocyte differentiation to protect against apoptosis [15,58]. Collectively, these findings provide compelling evidence of the beneficial roles of irisin in preserving musculoskeletal health under disuse conditions, suggesting irisin-based therapy as a potential approach against prolonged inactivity-induced musculoskeletal deterioration.

### 2.2. Myostatin

Myostatin, the first identified myokine expressed in developing and mature muscles, exerts a negative regulatory role in muscle development [59]. Evidence has suggested that mice deficient in myostatin [60,61,62,63], mice treated with the myostatin inhibitor MID-35 [64], mice treated with myostatin monoclonal antibodies [65], cynomolgus monkeys treated with myostatin antibody GYM329 [66], and myostatin knockout cattle [67,68,69], dogs [70], and pigs [71,72] all exhibit higher muscle mass, implying that reducing myostatin expression in varying mammalian species markedly promotes muscle growth. Further research on the underlying mechanisms has indicated that myostatin depletion promotes myoblast proliferation and differentiation and increases muscle mass by elevating the expression of myogenic regulatory factors, including myogenic differentiation antigen (MyoD), myogenin (MyoG), and myogenic factor-5 (Myf-5) [27,28], activating the PI3K/protein kinase B (PKB, Akt)/mammalian target of rapamycin (mTOR) signaling pathway [27,28,29] while inhibiting the SMAD 2/3 pathway (the canonical pathway of myostatin) [30]. These findings are in contrast to those observed upon myostatin overexpression, which are accompanied by the inhibition of protein synthesis [32] and myoblast differentiation [33]. These contrasting outcomes, viewed from different perspectives, further underscore the significant negative influence of myostatin in protein metabolism. In addition, evidence has demonstrated that reduced myostatin expression inhibits the SMAD 2/3 signaling pathway, promotes AMPK expression, enhances glucose-6-phosphate dehydrogenase (G6PD) enzyme activity, and increases skeletal muscle antioxidant capacity, suggesting negative regulatory roles regarding antioxidant capacity [31]. Thus, targeting myostatin may serve as an effective strategy to preserve muscle mass.

Myostatin exerts various negative regulatory effects on bone health. Research has shown an association between elevated blood concentrations of myostatin and reduced cortical bone thickness [73]. Conversely, myostatin deficiency in mice has been shown to lead to an increase in bone mineral content [60,61,63,74]. Mechanistic research has also indicated that the detrimental effects of myostatin on bone are closely related to the inhibition of osteoblast formation [34], disruption of osteoblast differentiation, weakened osteocyte function [35], and promotion of osteoclastogenesis [34,36], emphasizing the negative impacts of myostatin on bone via disturbance of the bone turnover process. Thus, myostatin may be an important therapeutic target in musculoskeletal disorders.

Elevated myostatin levels may also be involved in the occurrence of disuse-induced muscle atrophy. Evidence has shown that serum levels of myostatin in young human males are elevated on the second day of recovery after 10 days of bed rest [54]. Hindlimb unloading [75], immobilization [76], and spaceflight [77] are all associated with elevated myostatin mRNA expression in skeletal muscles. These findings demonstrate that myostatin levels are elevated in both the circulatory system and local muscle under conditions of disuse. In addition, the suppression of myostatin expression has been shown to rescue the deterioration of skeletal muscle structure and function under disuse conditions [78]. As such, targeting myostatin through genetic and pharmacological interventions may be an effective approach to preserve musculoskeletal quality during spaceflight [79]. However, research has indicated that a loss of myostatin function does not protect against a loss of iliopsoas mass in mice subjected to unloading [80], suggesting that the protective effects of myostatin knockout may not extend to all skeletal muscle types under disuse conditions, potentially due to the variations in location and function among these muscles.

### 2.3. Other Myokines

BAIBA is a relatively novel small molecule identified in the supernatant of cultured myocytes [81], which preserves skeletal muscle via the amelioration of insulin resistance and inflammation, playing a strong regulatory role in the metabolic and immune homeostasis of muscle cells [37]. BAIBA administration has also been shown to promote bone metabolism balance by stimulating osteoblast differentiation [38,82] and increase osteocyte viability by suppressing mitochondrial fission and preserving mitochondrial fusion [39,40]. The positive effects of BAIBA have also been verified under disuse conditions, with BAIBA supplementation found to alleviate muscle fiber type transition from type I to type II in soleus muscle by promoting peroxisome proliferator-activated receptor δ (PPARδ) expression, preserving skeletal muscle function, and maintaining proximal tibiae trabecular bone mass by attenuating osteocyte apoptosis in hindlimb-unloaded mice [39,40]. As such, BAIBA represents a promising molecular therapy for disuse-induced musculoskeletal deterioration.

Lumican is a small interstitial proteoglycan secreted by skeletal muscle cells [83], which promotes myogenesis by activating the P38 signaling pathway and maintains protein balance by up-regulating protein synthesis and down-regulating protein degradation [41]. The protective roles of lumican on bone have also been well established in both in vitro and in vivo studies. Lee et al. reported that lumican plays crucial roles in maintaining musculoskeletal metabolism, not only promoting bone anabolism by interacting with integrin α2β1 and activating the ERK signaling pathway [42], but also inhibiting osteoclastogenesis by suppressing Akt signals [43]. Further studies have shown a decrease in plasma lumican concentrations after 10 days of bed rest [84], while the administration of lumican partially prevents the reduction in muscle mass and muscle fiber cross-sectional area observed in muscles after two weeks of unloading [41], highlighting its role in protecting skeletal muscles. Notably, a recent proteomics analysis of astronauts observed that lumican protein expression in the soleus muscle is elevated after 11 days of spaceflight [85]. However, further verification experiments are needed to confirm the correlation between the elevation of lumican in muscle tissue and its decline in plasma under disuse conditions.

Initially identified as a myokine in 2003 [10], subsequent research has shown that the direct administration of IL-6 can result in lower muscle mass as well as total protein and myofibrillar protein content in skeletal muscle [44], which is closely linked to weakened myoblast differentiation, fusion, and muscle protein turnover, as evidenced by the suppression of protein synthesis and enhancement of protein degradation [45,86]. In addition to its negative regulatory effects on skeletal muscle, IL-6-transgenic mice exhibit a deterioration in skeletal structure [46], associated with a decrease in osteoblast differentiation and an increase in osteoclast activity [47,87]. Studies focusing on changes in IL-6 levels under disuse conditions have reported that circulating IL-6 levels are elevated in astronauts following short-duration spaceflight (10–15 days) [88], in healthy males after 14 days of bed rest [89], and in mice after two weeks of hindlimb unloading [90]. Moreover, IL-6 mRNA expression is increased in the skeletal muscles of humans after seven days of bed rest [91], in the gastrocnemius muscle of rats after seven days of hindlimb unloading [92], and in the gastrocnemius muscle of mice after 10 [93] or 14 days of immobilization [94]. The detrimental effects of IL-6 on the musculoskeletal system and its increase under disuse conditions suggested that elevated levels may be associated with musculoskeletal deterioration. Accordingly, subsequent studies have shown that IL-6 receptor inhibition can suppress muscle RING finger 1 (MuRF-1) expression, thereby preventing mechanical unloading-induced muscle atrophy [90]. However, whether such inhibition is also of benefit for bone preservation under disuse conditions requires further evaluation.

Collectively, myokines, such as irisin, BAIBA, and lumican, which play positive regulatory roles in skeletal muscles, also show beneficial roles in bone. The positive regulatory roles of these myokines on skeletal muscles are achieved by promoting muscle cell proliferation and differentiation, maintaining muscle protein metabolism balance, preserving mitochondrial function, and enhancing antioxidant and anti-inflammation capacity. Moreover, these myokines regulate bone metabolism by promoting bone formation, suppressing bone resorption, preserving osteocyte mitochondria, and inhibiting osteocyte apoptosis. Conversely, negative myokines, such as myostatin and IL-6, adversely regulate skeletal muscle maintenance, primarily by inhibiting muscle cell proliferation and differentiation, and disrupt skeletal muscle protein metabolism balance, primarily by suppressing bone formation and promoting bone resorption. Furthermore, the down-regulation of myokines that play a positive role in muscle and bone and the up-regulation of myokines that play a negative role in muscle and bone likely contribute to skeletal muscle atrophy and bone loss under disuse conditions. Regarding additional myokines like MOTS-c, a novel and bioactive mitochondrial-derived peptide [95], research has identified their protective role in the musculoskeletal system, including defense against metabolic stress in muscle [96] and involvement in the regulation of bone metabolism [97]. However, the literature detailing changes and regulatory mechanisms of MOTS-c under conditions of disuse remains scarce, indicating a gap that warrants further investigation.

## 3. Roles of Muscle-Derived EVs on Musculoskeletal Metabolism and Homeostasis under Normal and Disuse Conditions

EVs are nanovesicles packaged by a single membrane and can be broadly categorized into exosomes and microvesicles according to their biogenesis patterns [98,99]. Exosomes are generated within the endosomal system as intraluminal vesicles (ILVs) and are secreted upon the fusion of multivesicular endosomes (MVEs) with the cell surface, whereas microvesicles are formed by outward budding at the plasma membrane [100]. Although initially described as cellular waste carriers [101,102], these nanovesicles have since been discovered to contain a diverse array of components, including proteins, lipids, carbohydrates, mRNAs, and non-coding RNAs [103,104], which play significant roles in intercellular communication between donor and recipient cells in paracrine, autocrine, and endocrine manners [105,106,107,108,109,110].

Skeletal muscles, acting as important endocrine organs, are adept at secreting EVs, commonly referred to as muscle-derived EVs. Evidence suggests that these muscle-derived EVs not only facilitate intramuscular communication among similar cells within the muscle tissue [111,112] but also inter-organ communication, notably between muscle and bone [113,114,115,116]. Additionally, the contents of muscle-derived EVs play a crucial role in mediating interactions within muscle tissue and between muscle and bone. To date, however, research in this area remains somewhat limited, with existing studies primarily focusing on miRNAs [117,118,119,120]. In the following section, we discuss the roles of muscle-derived EVs, particularly communication mediated by miRNAs within skeletal muscle cells and between skeletal muscle and bone, and their impact on musculoskeletal deterioration under disuse conditions (Table 2).

### 3.1. Effects of Muscle-Derived EVs on Muscle

Following the initial discovery of EV secretion by C2C12 myoblasts [126], subsequent studies have revealed that muscle cells at various developmental stages, including myotubes and muscle progenitors, also possess this capability. These studies have validated the role of EVs and their molecular contents in mediating cell–cell communication and maintaining tissue homeostasis within skeletal muscles. Evidence has shown that EVs from myotubes contain 182 miRNAs, with miR-133a specifically playing a role in inhibiting myoblast proliferation and promoting their differentiation into myotubes by targeting and silencing Sirt1 expression [118]. Furthermore, upon absorption by myoblasts, EVs from myotubes, containing various proteins related to skeletal muscle contraction, can inhibit myoblastic proliferation by down-regulating the expression of cyclin-D1 and promote myogenic differentiation into myotubes by up-regulating myogenin [121]. In addition, exosomes from differentiated muscle cells enriched in multiple myogenic growth factors can stimulate MHC and desmin expression, thereby facilitating myogenesis [111]. Recent studies have found that EVs derived from skeletal muscle are predominantly distributed in the interstitial space [8,127], playing a crucial role in mediating homologous cell–cell communication within skeletal muscle tissue [119]. Research has reported that exosomal miR-1, -206, -431, and -486, derived from the muscle interstitium, can promote muscle differentiation by inhibiting the mRNA expression of paired box 7 (Pax-7) and promoting MHC expression [127]. These findings underscore the potential of EVs and their contents from differentiating muscle cells or tissues in driving cell differentiation and myogenesis. In addition, differentiated muscle-cell-derived EVs can also reduce collagen deposition and decrease fibrosis in skeletal muscle [111]. Fry et al. observed that exosomal miR-206 derived from muscle progenitors can also down-regulate collagen expression by inhibiting ribosome-binding protein 1 (Rrbp-1) expression [122], further demonstrating the important role of muscle-derived EVs in suppressing muscle fibrosis. Recent research has also highlighted the anti-inflammatory effects of myotube-derived EVs containing miR-206-3p, miR-378a-3p, miR-30d-5p, and miR-21a-5p, emphasizing the importance of muscle-derived EV miRNAs in maintaining the immune microenvironment [123]. Thus, muscle-derived EVs and their molecular contents, especially miRNAs, play essential positive roles in maintaining tissue homeostasis by promoting muscle cell differentiation, inhibiting muscle fibrosis, and combating inflammation.

### 3.2. Effects of Muscle-Derived EVs on Bone

Accumulating evidence has revealed the critical roles of muscle-derived EVs in regulating bone remodeling. Notably, myoblast-derived exosomal miR-27a-3p has been shown to promote MC3T3-E1 pre-osteoblast differentiation and bone mineralization by activating the Wnt/β-catenin signaling pathway [117]. Moreover, in differentiating C2C12 cells, exosomal paired-related homeobox 2 (Prrx2) promotes osteogenesis by alleviating the inhibitory effects of miR-128 on yes-associated protein 1 (YAP-1) via the up-regulation of long non-coding RNA (lncRNA)-MIR22HG expression [124]. Muscle-derived EVs can also promote osteogenic differentiation [9]. In addition to their positive effects on bone formation, EVs also play inhibitory roles in bone resorption. For example, miR-196a-5p participates in C2C12 myoblast- and myotube-derived EV suppression of osteoclast formation by reducing mitochondrial function [120,125]. Muscle-derived EVs also exert inhibitory effects on osteoclasts [9]. Thus, muscle-derived EVs play a positive role in bone metabolism via the promotion of bone formation and inhibition of bone resorption.

### 3.3. Changes and Effects of Muscle-Derived EVs under Disuse Conditions

Recent research has reported no notable differences in serum EV concentrations between normal and hindlimb-unloaded rats [8]. Van et al. observed an increase over time in the mRNA expression of transmembrane proteins related to EV formation, including CD63 and CD9, within the soleus and quadriceps muscle of rats subjected to tail suspension, but a significant down-regulation in the gastrocnemius muscle [128,129]. This variation in EV secretion patterns due to disuse may be associated with the specific characteristics of different skeletal muscle tissues.

An analysis of molecules within muscle-derived EVs has revealed that the expression levels of miRNAs associated with cellular senescence and muscle atrophy, such as miR-let-7c, miR-let-7b, miR-181a, and miR-124, are increased in fibro-adipogenic progenitor cell-derived EVs following 14 days of single-hindlimb immobilization in mice [130], which may be a potential mechanism involved in disuse-induced muscle atrophy. In addition, recent findings have indicated that skeletal muscle-derived EVs from mice treated with botulinum toxin or sciatic neurotomy can inhibit the formation of bone marrow stromal cells (BMSCs) and promote osteoclastogenesis [9], implying that muscle-derived EVs may contain more molecules harmful to bone growth, meriting further investigation. The same study also found that muscle-derived EVs from normal mice can significantly inhibit osteoclast differentiation, further emphasizing the important role of muscle-derived EVs in bone maintenance. Thus. screening key effector molecules and exploring their potential underlying mechanisms remain urgent research directions [9].

## 4. Conclusions and Future Directions

**Figure 1:**
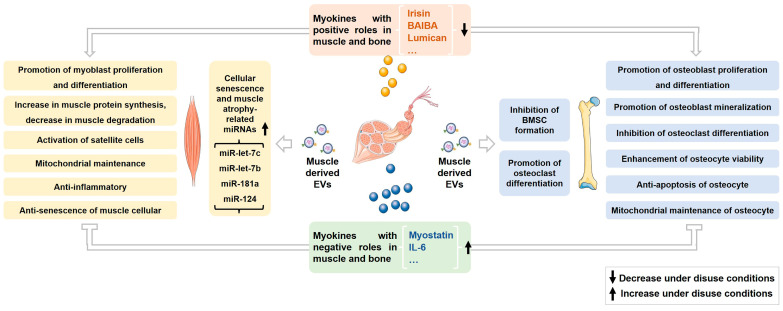
Overview of changes and regulatory roles of myokines and muscle-derived EVs on muscle and bone under disuse conditions.

Current research has thoroughly demonstrated the essential functions of skeletal muscle-derived myokines and EVs in controlling the homeostasis of both skeletal muscle and bone (Figure 1). These myokines and EVs are crucial for preserving the overall balance of the musculoskeletal system, influencing the proliferation and differentiation of muscle and bone cells, regulating musculoskeletal metabolism, and maintaining mitochondrial stability. The decrease in myokines beneficial for muscle and bone, coupled with the increase in myokines detrimental to these tissues, likely contributes to the degenerative changes observed in muscle and bone by disrupting musculoskeletal balance under conditions of disuse. Furthermore, evidence also suggests a close relationship between increased miRNAs in muscle-derived EVs, linked to cellular senescence and muscle atrophy, and muscle atrophy induced by inactivity. Nevertheless, research regarding additional mechanisms by which muscle-derived EVs may mediate bone loss under disuse conditions, particularly in vivo studies, remains limited. Employing targeted labeling of muscle-derived EVs with fluorescent probes and tracking their distribution in bone cells using techniques such as small animal imaging systems could establish a theoretical basis for a more comprehensive analysis of the mechanisms involved in muscle-derived EV-mediated bone loss during disuse. Such studies may offer new strategies for addressing disuse-induced osteoporosis by targeting specific effectors within muscle-derived EVs.

## Figures and Tables

**Table 1:** Regulation of muscle and bone by myokines.

Myokine		Target Cell/Tissue	Effect and Mechanism	References
Irisin		C2C12 myoblasts	Activates satellite cells Enhances protein synthesis through activating Akt/mTOR pathway and down-regulates protein degradation through suppressing protein expression of Atrogin-1 and MuRF-1	[12]
		C2C12 myoblasts	Enhances myoblast proliferation and fusion through up-regulating mRNA expression of ERK-dependent chemokine (C-C motif) ligand 7 (CCL-7)	[13]
		Human skeletal muscle cell	Stimulates muscle growth through up-regulating mRNA expression of IGF-1 and down-regulating mRNA expression of myostatin	[14]
		C2C12 myoblast	Preserves muscle cell from senescence through inhibiting mRNA expression of senescence marker, p53	[15]
		Hind muscle of female SD rats	Promotes mitochondrial fusion Increases mRNA expression of main regulatory genes for mitochondrial fusion, DPL1, and Mfn	[16]
		C2C12 myotubes	Increases mitochondrial content and oxygen consumption through up-regulating mRNA and protein expression of several genes including peroxisome proliferator-activated receptor gamma coactivator-1alpha (PGC-1α)	[17]
		BMSCs	Enhances osteoblast differentiation via increasing mRNA expression of Alp and Col-1	[18]
		BMSCs	Promotes osteogenesis through up-regulating mRNA expression of osteogenic markers, including Runx-2, bone sialoprotein (Bsp), Col-1, and Alp Promotes BMSCs mineralization Inhibits osteoclastogenesis through decreasing mRNA expression of osteoclastogenesis markers, including tartrate-resistant acid phosphatase (Trap), matrix metalloproteinase 9 (Mmp-9), and NFATc1	[19]
		Murine osteoblastic MC3T3-E1 cells	Promotes osteoblast proliferation and differentiation through activating P38/ERK MAPK signaling pathway	[20]
		MC3T3-E1 osteoblasts	Enhances osteogenic differentiation via increasing mRNA expression of osteogenic genes, Alp, Col-1, Runx-2, osterix, Opn, Ocn, Opg, and ERα	[21]
		MC3T3-E1 osteoblast precursor cells RAW264.7 osteoclast precursor cells	Increases osteoblastogenesis and mineralization through activating β-catenin signaling Inhibits RANKL-induced osteoclastogenesis through decreasing mRNA expression of nuclear factor of activated T-Cells, cytoplasmic 1 (NFATc1)	[22]
		Tibia of young male mice	Stimulates bone formation through up-regulating mRNA expression of Atf-4, Runx-2, Osx, low density lipoprotein receptor-related protein 5 (Lrp-5), β-catenin, Alp, and Col-1a1 Inhibits osteoclastogenesis and reduces osteoclast numbers	[23]
		MC3T3-E1 cells	Enhances M2 polarization of osteoblasts through activating AMPK signaling pathway	[24]
		Mouse bone marrow monocytes RAW264.7 cells	Promotes osteoclast precursor cell proliferation through activating p38 and JNK signaling pathway Inhibits differentiation of osteoclast cells through suppressing NF-κB pathway	[25]
		Osteocyte-like cells (MLO-Y4)	Prevents apoptosis of osteocyte-like cells (MLO-Y4)	[26]
Myostatin	Deletion	Luxi yellow cattle muscle	Promotes myogenic differentiation through activating PI3K/Akt/mTOR signaling pathway	[27]
		Longissimus dorsi of Liang Guang Small Spotted pigs	Promotes proliferation and myogenic differentiation of skeletal muscle cells through elevating protein expression of myogenic regulatory factors, MyoD, MyoG, and Myf-5	[28]
		Bovine skeletal muscle satellite cells (BSMSCs)	Promotes proliferation and myogenic differentiation of BSMSCs through increasing mRNA and protein expression of extracellular matrix and ribosome-related proteins, COL-1A1, activating focal adhesion, PI3K-Akt, and ribosomal pathways	[29]
		C2C12 myoblasts	Promotes C2C12 proliferation and differentiation through inhibiting myostatin canonical signaling pathway	[30]
		Bovine muscle	Enhances antioxidant capacity through activating SMAD-AMPK-G6PD signaling pathway	[31]
	Administration	C2C12 myoblasts	Inhibits protein synthesis through suppressing eukaryotic elongation factor 2 (eEF-2) through AMPK signaling pathway	[32]
		C2C12 myoblasts	Inhibits myoblast differentiation	[33]
		Primary mouse osteoblasts osteoclasts	Inhibits osteoblastic differentiation and mineralization through decreasing ALP activity, mRNA expression of osteoblast transcription factors osterix and Runx-2, as well as OCN secretion Promotes RANKL-induced osteoclastogenesis through increasing number of TRAP+ multinucleated giant cells, TRAP activity, and mRNA expression of NFATc1	[34]
		RANKL-induced osteoclasts Cultured osteocytic (Ocy454) cells	Inhibits osteoblastic differentiation through suppressing osteocyte-derived exosomal miR-218 Weakens osteocyte function via promoting mRNA expression of several bone regulators such as sclerostin (SOST), dickkopf-related protein 1 (DKK-1), and RANKL	[35]
		Bone marrow-derived macrophages (BMMs)	Promotes osteoclastogenesis through activating MAPK pathways and SMAD2 signaling	[36]
BAIBA		C2C12 cells	Attenuates insulin resistance and suppresses inflammation through activating AMPK–PPARδ signaling pathway	[37]
		MC3T3-E1 cells	Promotes proliferation and differentiation of osteoprogenitor cells through activating NAD(P)H oxidase/ROS signaling pathway	[38]
		Osteocytes	Increases osteocyte viability through blocking mitochondrial fission and preserving mitochondrial integrity	[39]
		Osteocytes	Prevents ROS induced mitochondria breakdown through activating Mas-related G protein-coupled receptor type D (MRGPRD)	[40]
Lumican		C2C12 myoblasts	Promotes myogenesis through activating p38 MAPK-mediated myoblast differentiation	[41]
		C2C12 myoblasts	Maintains positive protein balance through up-regulating protein synthesis and down-regulating protein degradation	[41]
		Murine preosteoblast MC3T3-E1 cells	Stimulates bone formation via integrin α2β1 and the downstream ERK signal	[42]
		Primary bone marrow cells	Inhibits osteoclastogenesis and bone resorption through suppressing Akt activity	[43]
IL-6		TA and EDL muscles of rats	Decreases total protein and myofibrillar protein content through decreasing phosphorylation of ribosomal S6 kinase and signal transducers and activators of transduction 5 (STAT-5)	[44]
		Skeletal muscle of mice	Inhibits basal protein synthesis through suppressing mTORC1 signaling	[45]
		Primary osteoblasts and osteoclasts of mice	Decreases osteoblast and increases osteoclast number and activity	[46]
		MC3T3-E1 osteoblastic cells	Negatively regulates osteoblast differentiation through activating Src-homology domain 2 containing protein-tyrosine phosphatase (SHP-2)/mitogen-activated protein kinase-extracellular signal–regulated kinase kinase (MEK-2)/ERK and SHP-2/PI3K/Akt-2 pathways, as well as reducing mRNA expression of osteoblastic differentiation related genes, including Alp, Runx-2, and Ocn	[47]

**Table 2:** Regulation of muscle and bone by muscle-derived EVs.

Muscle-Derived EVs Containing miRNAs	Target Cell/Tissue	Effect and Mechanism	References
C2C12 myotube-derived exosomal miR-133a	C2C12 myoblasts	Inhibits myoblast proliferation and promotes myoblast differentiation into myotube through silencing Sirt-1	[118]
C2C12 myotube-derived exosomal proteins	C2C12 myoblasts	Inhibits myoblast proliferation through down-regulating mRNA expression of cyclin-D1 Promotes myoblast differentiation into myotubes through up-regulating mRNA expression of MyoG	[121]
Exosomes released from differentiating human skeletal myoblasts	Human adipose-derived stem cells Hindlimb muscles of mice	Promotes myogenesis through increasing expression of myogenic proteins (myosin heavy chain and desmin) Alleviates skeletal muscle fibrosis through reducing collagen deposition	[111]
Muscle interstitium-derived exosomal miR-1, -206, -431, and -486	C2C12 myoblasts	Promotes muscle differentiation through inhibiting mRNA expression of Pax-7 and promotes mRNA expression of MHC	[112]
Myogenic progenitor cell-derived exosomal miR-206	Extracellular matrix	Inhibits excessive extracellular matrix generation through suppressing protein expression of Rrbp-1 and down-regulates mRNA expression of collagen proteins involved in biosynthesis	[122]
miR-206-3p, miR-378a-3p, miR-30d-5p, and miR-21a-5p in myotube-derived EVs	Mouse bone marrow-derived macrophages	Exhibits anti-inflammatory effects in macrophages through activating PI3K-Akt and JAK-STAT pathways	[123]
Myoblast-derived exosomal miR-27a-3p	MC3T3-E1 pre-osteoblasts	Promotes MC3T3-E1 pre-osteoblast differentiation and bone mineralization through activating Wnt/β-catenin signaling pathway	[117]
Differentiating C2C12 cell-derived exosomal Prrx-2	BMSCs	Promotes osteogenesis differentiation through alleviating inhibitory effects of miR-128 on YAP-1 via up-regulating lncRNA MIR22HG	[124]
Skeletal muscle-derived EVs	Primary BMSCs and osteoclasts of C57BL/6J mice	Promotes osteogenesis differentiation of BMSCs through inhibiting osteoclast formation	[9]
C2C12 myoblast- and myotube-derived EV miR-196a-5p	Raw264.7 cells	Suppresses osteoclast formation through weakening mitochondrial function of osteoclasts	[120,125]

## Data Availability

Not applicable.

## Abbreviations

ALP	alkaline phosphatase protein
AMPK	AMP-activated protein kinase
BAIBA	β-aminoisobutyric acid
BMP	bone morphogenetic protein
BMSCs	bone marrow stromal cells
Bsp	bone sialoprotein
CCL-7	chemokine (C-C motif) ligand 7
COL-1	collagen I
DKK-1	dickkopf-related protein 1
eEF-2	eukaryotic elongation factor 2
Erα	estrogen receptor alpha
ERK	extracellular signal-regulated kinase
EVs	extracellular vesicles
FNDC-5	fibronectin type III domain-containing 5
G6PD	glucose-6-phosphate dehydrogenase
IGF-1	insulin-like growth factor 1
IL-6	interleukin 6
ILVs	intraluminal vesicles
JNK	c-Jun N-terminal kinase
Lrp-5	low density lipoprotein receptor-related protein 5
MAPK	mitogen-activated protein kinase
MEK-2	mitogen-activated protein kinase-extracellular signal-regulated kinase
MHC	myosin heavy chain
miRNAs	microRNAs
Mmp-9	matrix metalloproteinase 9
MRGPRD	mas-related G protein-coupled receptor type D
mTOR	mammalian target of rapamycin
MuRF-1	muscle RING finger 1
MVEs	multivesicular endosomes
Myf-5	myogenic factor-5
MyoD	myogenic differentiation antigen
MyoG	myogenin
NFATc1	nuclear factor of activated T-Cells, cytoplasmic 1
NF-κB	nuclear factor kappa-B
OCN	osteocalcin
OPG	osteoprotegerin
OPN	osteopontin
Pax-7	paired box 7
PGC-1α	peroxisome proliferator-activated receptor gamma coactivator-1alpha
PI3K	phosphatidylinositol 3-kinase
PKB, Akt	protein kinase B
PPARδ	peroxisome proliferator-activated receptor δ
RANK	receptor activator of nuclear factor-κ-gene binding
Rrbp-1	Ribosomal binding protein 1
RUNX-2	runt-related transcription factor 2
SHP-2	Src-homology domain 2 containing protein-tyrosine phosphatase
SOST	sclerostin
STAT-5	signal transducers and activators of transduction 5
Trap	tartrate-resistant acid phosphatase
YAP-1	yes-associated protein 1
